# Supplementary material for: Pandemic buying: Testing a psychological model of over-purchasing and panic buying using data from the United Kingdom and the Republic of Ireland during the early phase of the COVID-19 pandemic
Source: PLoS One. 2021 Jan 27;16(1):e0246339. doi: 10.1371/journal.pone.0246339 (PMC7840055; doi:10.1371/journal.pone.0246339)
Supplement: S1 Table — (DOCX) [file pone.0246339.s001.docx]

S1 Table: Sociodemographic characteristics of the Irish and UK samples (reproduced from Murphy et al. [1]

| Ireland (N = 1041) | % | UK (N = 2025) | % |
| --- | --- | --- | --- |
| **Sex** |  | **Sex** |  |
| Female | 51.5 | Female | 51.7 |
| Male | 48.2 | Male | 48.3 |
| **Age** |  | **Age** |  |
| 18-24 | 11.1 | 18-24 | 12.1 |
| 25-34 | 19.2 | 25-34 | 18.8 |
| 35-44 | 20.6 | 35-44 | 17.4 |
| 45-54 | 15.9 | 45-54 | 20.2 |
| 55-64 | 21.0 | 55-64 | 17.2 |
| 65+ | 12.2 | 65+ | 14.2 |
| **Birthplace** |  | **Birthplace** |  |
| Ireland | 70.7 | UK | 90.6 |
| **Region of Ireland** |  | **Region of UK** |  |
| Leinster | 55.3 | England | 86.9 |
| Munster | 27.3 | Scotland | 7.8 |
| Connaught | 12.0 | Wales | 3.1 |
| Ulster | 5.4 | Northern Ireland | 2.3 |
| **Ethnicity** |  | **Ethnicity** |  |
| Irish | 74.8 | White British/Irish | 85.5 |
| Irish Traveller | 0.3 | White non-British/Irish | 5.7 |
| Other White background | 17.3 | Indian | 2.0 |
| African | 1.9 | Pakistani | 1.3 |
| Other Black background | 0.3 | Chinese | 0.9 |
| Chinese | 0.4 | Afro-Caribbean | 0.6 |
| Other Asian | 3.2 | African | 1.3 |
| Mixed Background | 1.8 | Arab | 0.1 |
|  |  | Bangladeshi | 0.3 |
|  |  | Other Asian | 0.5 |
| **Living location** |  | **Living location** |  |
| City | 24.5 | City | 24.6 |
| Suburb | 18.1 | Suburb | 28.2 |
| Town | 26.8 | Town | 30.6 |
| Rural | 28.8 | Rural | 16.5 |
| **Highest Education** |  | **Highest Education** |  |
| No qualification | 1.2 | No qualifications | 2.9 |
| Finished mandatory schooling | 6.4 | O-level/GCSE or similar | 19.0 |
| Finished secondary school | 22.4 | A-level or similar | 18.1 |
| Undergraduate degree | 22.5 | Diploma | 5.6 |
| Postgraduate degree | 19.8 | Undergraduate degree | 28.2 |
| Other technical qualification | 27.9 | Postgraduate degree | 15.6 |
|  |  | Technical qualification | 9.3 |
|  |  | Other | 1.3 |
| **2019 income** |  | **2019 income** |  |
| 0-€19,999 | 24.6 | £0-£15490 | 20.2 |
| €20,000-€29,999 | 21.3 | £15,491-£25,340 | 20.2 |
| €30,000-€39,999 | 19.5 | £25,341-£38,740 | 19.0 |
| €40,000-€49,999 | 12.7 | £38,741-£57,930 | 20.2 |
| €50,000+ | 21.9 | £57,931+ | 20.2 |
| **Employment status** |  | **Employment status** |  |
| Full-time (self)/employed | 43.3 | Full-time (self)/employed | 48.8 |
| Part-time (self)/employed | 15.7 | Part-time (self)/employed | 15.0 |
| Retired | 15.0 | Retired | 16.5 |
| Unemployed | 8.4 | Unemployed | 11.7 |
| Student | 6.3 | Student | 4.7 |
| Unemployed (disability or illness) | 5.6 | Unemployed (disability or illness) | 3.4 |
| Unemployed due to COVID-19 | 5.7 |  |  |
| **Religion** |  | **Religion** |  |
| Christian | 69.8 | Christian | 50.4 |
| Muslim | 1.6 | Muslim | 3.0 |
| Jewish | 0.2 | Jewish | 0.8 |
| Hindu | 1.1 | Hindu | 0.6 |
| Buddhist | 0.6 | Buddhist | 0.8 |
| Sikh | 0.1 | Sikh | 0.5 |
| Other religion | 3.8 | Other | 6.0 |
| Atheist | 15.3 | Atheist | 25.4 |
| Agnostic | 7.5 | Agnostic | 12.5 |
| **Lone adult in household** |  | **Lone adult in household** |  |
| Yes | 18.4 | Yes | 22.4 |
| **Children in the household** |  | **Children in the household** |  |
| Yes | 39.7 | Yes | 29.2 |

[1] Murphy J, Vallieres F, Bentall RP, Shevlin M, McBride O, Hartman TK, et al. Psychological characteristics associated with COVID-19 vaccine hesitancy and resistance in Ireland and the United Kingdom. Nat Commun. 2021;12:29.
