# Supplementary material for: Pandemic buying: Testing a psychological model of over-purchasing and panic buying using data from the United Kingdom and the Republic of Ireland during the early phase of the COVID-19 pandemic
Source: PLoS One. 2021 Jan 27;16(1):e0246339. doi: 10.1371/journal.pone.0246339 (PMC7840055; doi:10.1371/journal.pone.0246339)
Supplement: S2 Table — (DOCX) [file pone.0246339.s002.docx]

S2 Table: Correlation matrix of predictor variables.


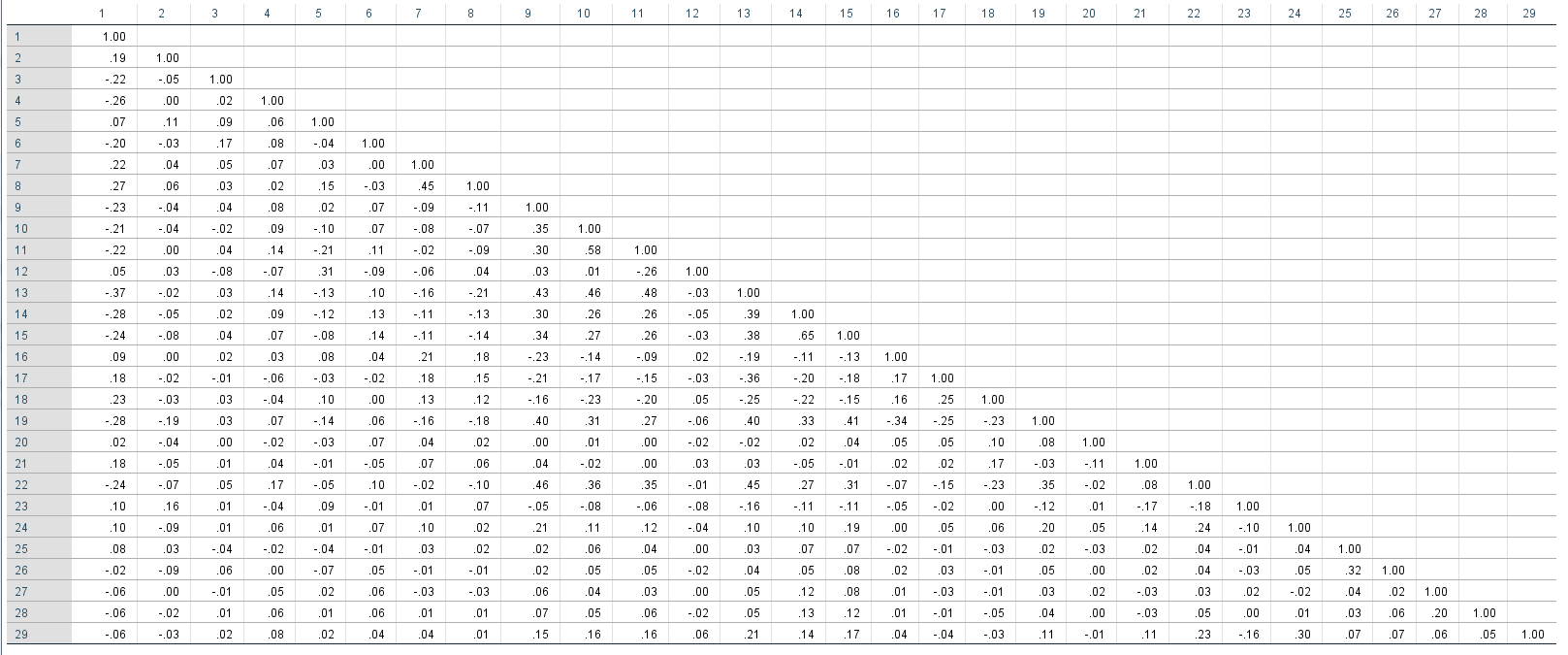


Note: The corresponding critical correlation value (*r_c_*​) for a significance level of alpha = .05 for a two-tailed test is *r_c_*​= .035

Key to S2 Table

1. Age,
2. Gender (male)
3. Number adults
4. Number children
5. Income
6. Lost income
7. Neighbourhood belonging
8. Neighbourhood Trust
9. Intolerance of Uncertainty
10. LOC: Chance
11. LOC: Powerful Others
12. LOC: Internal
13. Paranoia
14. Depression (>10)
15. Anxiety (>10)
16. Extraversion
17. Agreeableness
18. Conscientiousness
19. Neuroticism
20. Openness
21. Right Wing Authoritarianism
22. Death Anxiety
23. CRT
24. COVID-19 Anxiety
25. Health problems - self
26. Health problems - other
27. Perceived infection status - self
28. Perceived infection status - other
29. Personal Risk
